# Reply to the editor: interpreting MAPSTROKE capacity modelling in the context of bottlenecks across the stroke care pathway

**DOI:** 10.1093/esj/aakag021

**Published:** 2026-03-23

**Authors:** Ettore Nicolini, Antonio Ciacciarelli, Leonardo Augusto Carbonera

**Affiliations:** Department of Human Neuroscience, Sapienza University of Rome, Rome, Italy; Stroke Unit, Policlinico Umberto I, Sapienza University of Rome, Rome, Italy; Department of Neurology and Neurosurgery, Hospital Moinhos de Vento, Porto Alegre-RS, Brazil; Pós-Graduação em Medicina: Ciências Médicas, Universidade Federal do Rio Grande do Sul, Porto Alegre-RS, Brazil

**Keywords:** algorithms, geographic information systems, health care planning, health services accessibility, medical informatics, stroke

Dear Editor,

We thank Drs Merlino, Gigli and Valente for their letter regarding our recently published manuscript, “The MAPSTROKE analysis of the access to stroke reperfusion treatment and stroke units in Italy.”^1^

In their correspondence “Stroke unit capacity should not rely on shortening length of stay alone”, the authors appropriately highlight that, although reducing stroke unit (SU) length of stay (LOS) may increase bed turnover, this approach must not lead to premature transitions of care driven by bed pressure, particularly when downstream pathways are insufficient.^2^

We fully agree and appreciate the opportunity to further clarify how LOS was handled in our modelling and how our findings should be interpreted within the stroke care continuum in Italy and in other countries where the model may be applied.

First, we acknowledge the limited literature establishing universal thresholds for modelling SU throughput. In our analysis, we used each hospital’s reported LOS to estimate capacity, and we performed an additional scenario analysis using an “ideal” LOS of 3.65 days, derived from the European Stroke Organisation benchmark of 100 stroke patients per SU bed per year (365/100 = 3.65).^3^

This “ideal” value should not be interpreted as a recommended discharge target for all patients, but rather as a standardised benchmark for sensitivity analysis and international comparability. MAPSTROKE does not aim to define the optimal LOS, but rather to assess how different LOS scenarios influence system capacity under varying organisational conditions. In fact, even under the “ideal” LOS scenario, the model still identified a substantial gap in SU bed capacity. Consistent with the concerns raised by Merlino et al.^2^, LOS is not merely a modifiable efficiency parameter. Observed LOS may reflect genuine clinical needs, as well as downstream organisational bottlenecks that modelling did not fully capture in this publication.

Second, the mechanism that causes prolonged stays is often a system-level bottleneck: Patients may remain in SU beds longer than clinically necessary for intensive monitoring because stroke ward capacity, inpatient rehabilitation availability or community-based post-acute services are insufficient. In such settings, SU beds can function as a hybrid space for acute and subacute care, limiting access for incoming acute stroke patients. This effect was accurately described by Merlino et al.^2^

Importantly, the paper by Cadilhac et al.^4^, cited by Merlino et al.^2^, illustrates how LOS metrics should be interpreted within the broader care pathway. In that registry analysis, patients who spent 90% or more of their admission in the SU had a median hospital LOS of 4 days (approximately 3.6 days in the SU itself). However, only about 50%−55% were discharged home; the remainder required transfer to inpatient rehabilitation, another hospital ward or residential aged care.^4^ This reinforces that achieving shorter SU/hospital LOS in a safe and effective way is closely linked to the availability and organisation of structured post-acute services and continued care.

Finally, we agree that the interpretation of our results must explicitly incorporate the stroke care continuum. Reducing SU LOS toward benchmark values may be feasible in some contexts, but only when post-acute pathways (step-down care, early rehabilitation, early supported discharge and long-term care options) are adequately organised to ensure continuity of multidisciplinary stroke care after the SU phase.

The results of the Italian application of the MAPSTROKE computation strategy should, therefore, not be interpreted as advocating for shorter SU stays per se, but as highlighting that, even where LOS can be safely reduced through better organisation of post-stroke care pathways, additional SU bed capacity will still be required to meet the current burden of stroke. Our findings must, therefore, be interpreted as a tool for data-driven planning. Policy decisions should also consider the continuum of stroke care, thereby optimising specialised stroke pathways.

## Data Availability

Not applicable.
